# Golf and Binocular Vision

**Published:** 1907-07-06

**Authors:** W. C. Rivers


### The Practitioner's Relaxations and Hobbies.

### GOLF AND BINOCULAR VISION.

By W. C. RIVERS, M.R.C.S., L.R.C.P.(Lond.).

As far as our good friends the laity will allow us, we doctors look for our recreation a great deal to golf. It is a game which can be played at other than stated times, and it does not demand any high standard of physical fitness or training. Perhaps the medical profession has not, as in the case of other games, produced many performers of note, except at least the late Dr. A. J. T. Allan. But if, as one may hope it will not, golf were to oust football from its position as the favourite game of medical students, no doubt the standard of medical golf (not that it is low) would rise.

Someone—a Dr. Eddowes, I think—once tried to show in *Golf Illustrated* a connection between the optical blind spot and the missing of short putts. Here is another bit of applied physiology which has had up to now but small notice. Consider first what would happen if a right-handed golfer with a full, free swing, were suddenly to shut his left eye when he was at the limit of his upward movement, that is, just before he began to come down on the ball. Clearly the ball would be quite lost to him. For at some part of a good swing, with plenty of body-turn, the bridge of the nose is bound to come between the right eye and the ball: in other words, since one begins the swing with both eyes on the ball, there is involved a change (in the middle of the stroke too) from binocular to monocular vision. When the coaches impress the first maxim of the game on their pupils, they forget that they themselves break the letter of it every time they drive. A revised edition for those with full swings would, therefore, be (for right-handers) "keep your *left* eye on the ball." Now in most people the right is the master eye. For those whose physiology is as rusty as was that of the author, it may be said that the way to find out whether one is right- or left-eyed is to look with both eyes at some small distant object, and then to hold a key at arm's length, so that the object chosen is seen fairly within the hole in the key-handle. In most cases, on closing the right eye, the object will seem to jump away to the right. If it does, the right is the master eye; if not, the left is. This may be confirmed by squinting down one's nose with both eyes: the side of the nose which is better seen is also the side of the master eye. Now, the practical effect of suddenly cutting off the vision of the master eye, of changing rapidly from binocular vision to vision of the subordinate eye only, is in non-technical language, to cause a considerable upset.

But it may be said all this is assumption: no one with a reputation for sobriety ever described similar apparent movements of a golf-ball. And as to the key-ring experiment, if the object jumped out of the ring when the master-eye was closed, why it jumped back just as quickly when this was opened again. The right eye soon gets sight of the ball again, on the down swing; we know that visual impressions may persist after removal of the stimulus: there may thus be no real loss of binocular vision at all. One or two things make one feel inclined to doubt this reasoning. In the first place there are the great number of shortened swings one sees. A good many men use only half or three-quarter swings. If they have good physical strength not very much is lost thereby: a finalist in this year's amateur championship is described as having only a half-swing. But this good golfer took up the game late, it may be said; lack of boyish elasticity may have something to do with his method. Well, if so, why do many of these players clip their swing only when they have the ball before them, not in light-hearted preliminary practice at daisy-heads? Probably partly because, in order not to spoil all their adjustments, they have at all costs to let their master-eye cleave to the ball. Then there is the way in which most people swing and stand. The common thing, of course, is to stand well behind one's ball so as to swing over rather than away from it, perpendicularly rather than horizontally. And the common thing is generally the easy thing; all first-class players, on the other hand, in reality swing horizontally, or so Mr. Hilton says. Now it is simple to convince oneself by experiment that the right eye comes off the ball sooner when one swings away from it than when one swings over it. Is this one reason why a horizontal swing is hard of accomplishment and therefore rare?

Better evidence is independent proof of a converse case—that a change from monocular to binocular vision is disadvantageous in the case of another ball-game. Mr. Spofforth, the famous Australian bowler, in *C. B. Fry's Magazine*, said that the batsmen he liked to bowl to were what he called "one-eyed" batsmen, those, that is, who presented their profile to the bowler and had in consequence only one eye on the ball as it came up the pitch. They were got rid of so easily that Mr. Spofforth, rather reluctantly giving away a bowler's secret, laid it down that all batsmen should face well round to the bowler, clap both eyes on the ball, and keep them there. Now, in the light of the foregoing, it is easy to see what happened to these foolish "one-eyed" batsmen. As most of them were right-handed, it was the left eye, the one which is generally the subordinate one, which fixed the ball as it left the bowler's hand. When it drew near and the batsman framed to make his stroke, both eyes were used. The master eye (the right) took on the duty of fixing; the left eye then adapted its line of vision to that of the other; the ball, therefore, may have seemed to "jump," as our object in the key-handle did when the master-eye was opened again—clearly an awkward thing to happen in the case of a bowler of Mr. Spofforth's pace. For the right-handed golfer, or cricketer, the condition of being right-eyed is probably a slight handicap, but is doubtless fairly easily overcome by aptitude or training. Perhaps certain fine drivers are right-handed and left-eyed. [*To be concluded.*]

